# UMPred-FRL: A New Approach for Accurate Prediction of Umami Peptides Using Feature Representation Learning

**DOI:** 10.3390/ijms222313124

**Published:** 2021-12-04

**Authors:** Phasit Charoenkwan, Chanin Nantasenamat, Md Mehedi Hasan, Mohammad Ali Moni, Balachandran Manavalan, Watshara Shoombuatong

**Affiliations:** 1 Modern Management and Information Technology, College of Arts, Media and Technology, Chiang Mai University, Chiang Mai 50200, Thailand; phasit.c@cmu.ac.th; 2 Center of Data Mining and Biomedical Informatics, Faculty of Medical Technology, Mahidol University, Bangkok 10700, Thailand; chanin.nan@mahidol.edu; 3 Tulane Center for Biomedical Informatics and Genomics, Division of Biomedical Informatics and Genomics, John W. Deming Department of Medicine, School of Medicine, Tulane University, New Orleans, LA 70112, USA; mhasan1@tulane.edu; 4 Artificial Intelligence & Digital Health Data Science, School of Health and Rehabilitation Sciences, Faculty of Health and Behavioural Sciences, The University of Queensland, St Lucia, QLD 4072, Australia; m.moni@uq.edu.au; 5 Department of Physiology, Ajou University School of Medicine, Suwon 16499, Korea

**Keywords:** umami peptide, sequence analysis, bioinformatics, machine learning, feature representation learning

## Abstract

Umami ingredients have been identified as important factors in food seasoning and production. Traditional experimental methods for characterizing peptides exhibiting umami sensory properties (umami peptides) are time-consuming, laborious, and costly. As a result, it is preferable to develop computational tools for the large-scale identification of available sequences in order to identify novel peptides with umami sensory properties. Although a computational tool has been developed for this purpose, its predictive performance is still insufficient. In this study, we use a feature representation learning approach to create a novel machine-learning meta-predictor called UMPred-FRL for improved umami peptide identification. We combined six well-known machine learning algorithms (extremely randomized trees, k-nearest neighbor, logistic regression, partial least squares, random forest, and support vector machine) with seven different feature encodings (amino acid composition, amphiphilic pseudo-amino acid composition, dipeptide composition, composition-transition-distribution, and pseudo-amino acid composition) to develop the final meta-predictor. Extensive experimental results demonstrated that UMPred-FRL was effective and achieved more accurate performance on the benchmark dataset compared to its baseline models, and consistently outperformed the existing method on the independent test dataset. Finally, to aid in the high-throughput identification of umami peptides, the UMPred-FRL web server was established and made freely available online. It is expected that UMPred-FRL will be a powerful tool for the cost-effective large-scale screening of candidate peptides with potential umami sensory properties.

## 1. Introduction

In foods, sensory flavor is closely connected with food selection, consumption, absorption, and digestion [1]. Although the umami taste has long been perceived in many traditional foods such as soy sauce, cheese, and fermented Asian foods, it was only recently that this taste quality was officially recognized [2]. The term “umami” is derived from the Japanese word (うま味), which means “pleasant savory taste”, feeling of “mouthfulness”, or deliciousness. In 2002, umami was identified as the fifth basic taste (after salty, sweet, sour, and bitter) to describe a pleasant savory or MSG-like flavor [3]. As a result, understanding the biophysical and biochemical properties of the umami taste is critical in both scientific research and the food industry. Because of the potential of umami peptides in the food industry, identifying and characterizing peptide umami intensity could be highly useful in both scientific and nonscientific research.

Several experimental methods, including reversed-phase high-performance liquid chromatography (RP-HPLC) and MALDI-TOF-MS analysis, have been used to identify and characterize peptides with umami sensory properties thus far [4,5]. To date, next-generation sequencing has resulted in the discovery of a large number of novel proteins, and it is possible that unknown candidate peptides from these proteins exhibit umami sensory properties. The existing experimental methods, however, are time-consuming and expensive. As a result, developing accurate and efficient computational methods for identifying umami peptides is necessary and can be a good complement to experimental methods. Several previous studies have concentrated on the identification and characterization of umami peptides, using computer-assisted methods such as homology modeling and molecular docking [6,7,8,9]. Meanwhile, the development of machine learning (ML)-based predictors could be useful in identifying umami-sensing peptides from large-scale protein sequences. Charoenkwan et al. recently developed iUmami-SCM [10], a novel sequence-based predictor. To the best of the authors’ knowledge, the reported iUmami-SCM can predict and analyze peptides with umami sensory properties based on sequence information, without knowing the 3D structure of the protein. The iUmami-SCM tool, in particular, was developed using a simple and interpretable scoring card method (SCM) in conjunction with estimated propensity scores of 20 amino acids and 400 dipeptides. Although this method has been used for the development of ML-based predictors of umami peptides with good performance as deduced from balanced accuracy (BACC), sensitivity (Sn), and Matthews coefficient correlation (MCC) of 0.824, 0.714, and 0.679, respectively, its overall prediction performance is not yet satisfactory enough owing to the inclusion of insufficient informative features and the use of only a single encoding and ML classifier.

**Figure 1:**
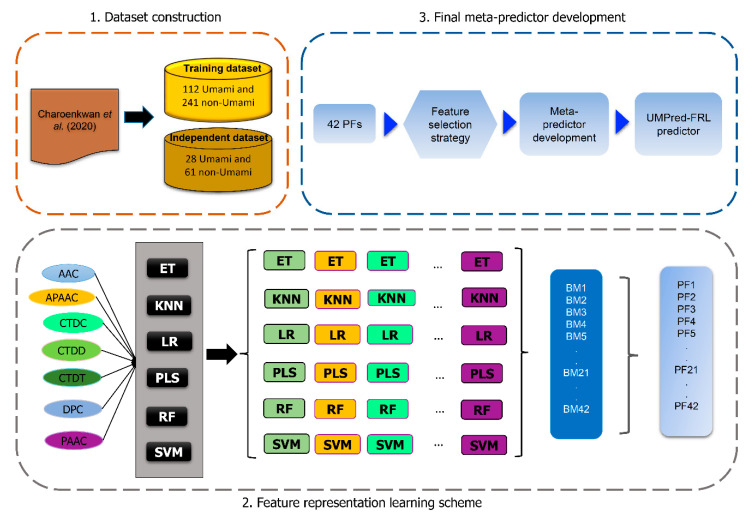
The overall flowchart of the development of UMPred-FRL. It consists of dataset construction, feature extraction, baseline model construction, new feature representation generation, and a final meta-predictor development.

Addressing the aforementioned issues, we present UMPred-FRL, a novel machine-learning meta-predictor that uses a feature representation learning method to improve the predictive performance of umami peptides. In the development of UMPred-FRL, we explored comprehensive and efficient feature encodings with popular ML algorithms. As we can see in Figure 1, we combined six different ML algorithms (extremely randomized trees (ET), k-nearest neighbor (KNN), logistic regression (LR), partial least squares (PLS), random forest (RF), and support vector machine (SVM)) with seven different feature encodings (amino acid composition (AAC), amphiphilic pseudo-amino acid composition (APAAC), dipeptide composition (DPC), composition (CTDC), transition (CTDT), distribution (CTDD), and pseudo-amino acid composition (PAAC)) for generating 42 baseline models. The predicted probabilistic scores of umami peptides were then estimated using these baseline models, and these new feature representations were considered. A final SVM-based meta-predictor was then developed by combining and selecting these new feature representations. On the basis of cross-validation and independent test datasets, our comparative results showed that UMPred-FRL outperformed its constituent baseline models. As for the independent test dataset, UMPred-FRL consistently outperformed the existing method (iUmami-SCM) in terms of BACC (0.860 vs. 0.824), Sn (0.786 vs. 0.714), and MCC (0.735 vs. 0.679). These findings demonstrated the proposed model’s efficacy and generalizability. Furthermore, our feature analysis revealed that when compared to seven well-known feature encodings, our proposed new feature representations had a higher discriminative capability to capture the key information about umami peptides. Finally, in order to maximize the utility of our proposed predictor, we created a publicly accessible web server at http://pmlabstack.pythonanywhere.com/UMPred-FRL (accessed on 1 December 2021). We believe that UMPred-FRL’s superior performance will allow for the rapid screening of candidate peptides with potential umami sensory properties.

## 2. Materials and Methods

### 2.1. Datasets

To ensure a fair comparison, the same benchmark datasets (UMP-TR and UMP-IND) presented in previous work were used to train and evaluate our proposed predictor [10]. This dataset contains 140 umami peptides and 304 non-umami peptides, which are categorized as positive and negative samples, respectively. Specifically, the positive samples were experimentally validated umami peptides identified in the literature [11,12,13,14,15,16] and the BIOPEP-UWM databases [17], while the negative samples were bitter peptides derived from our previous study [18]. All peptide sequences were unique in both positive and negative datasets. The UMP-TR dataset had 112 umami and 241 non-umami peptides, whereas the UMP-IND dataset had 28 umami and 61 non-umami peptides. These two datasets are available for free download at http://pmlabstack.pythonanywhere.com/UMPred-FRL (accessed on 1 December 2021).

### 2.2. Overall Framework of UMPred-FRL

Figure 1 depicts the overall development framework of UMPred-FRL. In particular, the illustration depicts the four main steps in the development of UMPred-FRL: feature extraction, baseline model construction, new feature representation generation, and final meta-predictor development. First, we used seven different feature descriptors from various perspectives (AAC, APAAC, CTDC, CTDD, CTDT, DPC, and PAAC). Second, using six well-known ML algorithms, these feature descriptors were used to create a pool of baseline models. Afterwards, by using the feature representation learning method [19,20,21], each baseline model was trained and used to generate new feature representations having class and probabilistic information. Finally, a set of new feature representations was combined to create a final meta-predictor.

### 2.3. Feature Encoding

We investigated the informative patterns of umami peptides using seven different encoding schemes, including AAC, APAAC, CTDC, CTDD, CTDT, DPC, and PAAC. These seven encoding schemes take into account twenty different types of 20 amino acids (A, C, D, E, F, G, H, I, K, L, M, N, P, Q, R, S, T, V, W, and Y) in peptide sequences and represent them in various N-dimensional (D) feature vectors. The seven encoding schemes are described in the subsections that follow.

#### 2.3.1. AAC and DPC

The frequency of 20 amino acids and 400 dipeptides is calculated using the AAC and DPC encoding schemes. These two encoding schemes have been used successfully to investigate a variety of protein and peptide functions. AAC and DPC provide 20D and 400D feature vectors for a given peptide sequence, respectively, and they are calculated as follows:

(1) $$f\left(i\right)=\frac{N\left(i\right)}{L},\;r\in \left\{A,\;C,\;D,\;E,\;F,\;G,\;H,\ldots ,Y\right\}$$

(2) $$f\left(i,j\right)=\frac{N\left(i,j\right)}{L-1},\;r,s\in \left\{A,\;C,\;D,\;E,\;F,\;G,\;H,\ldots ,Y\right\}$$

where *N*(*i*) is the frequency of amino acid represented by residue type © and *L* is the length of the peptide. Furthermore, *N*(*i,j*) is the frequency of dipeptide represented by residue types *i* and *j*.

#### 2.3.2. CTDC, CTDD and CTDT

Dubchak et al. developed the composition, transition, and distribution (CTD) method to predict protein folding class [22]. The three descriptors of composition (C), transition (T), and distribution (D) can be calculated using two factors: (i) Amino acid sequences which can be divided into specific structural sequences or by physicochemical properties of residues; and (ii) Tomii and Kanehisa’s main amino acid index [23] that is based on twenty amino acids, which have been divided into three groups on the basis of 13 different physicochemical properties including hydrophobicity, normalized van der Waals volume, polarity, polarization, charge, secondary structure, and solvent availability [24]. As a result, the percentage composition of each group in the peptide sequence has been described using these three descriptors. The work of Xiao et al. [19] provides more information on the characteristics of CTDC, CTDD, and CTDT. The iFeature module in the Python environment was used to construct three different types of sequence functions [20]. In particular, CTDC and CTDD can be calculated as follows:

(3) $$C\left(r\right)=\frac{N\left(r\right)}{L},\;r\in \left\{NE,\;PO,\;HY\right\}$$

(4) $$T\left(r,s\right)=\frac{N\left(r,s\right)+N\left(s,r\right)}{L-1},\;r,s\in \left\{\left(NE,\;HY\right),\left(PO,\;NE\right),\;\left(\;HY,\;PO\right)\right\}$$

(5) $$D\left(r\right)=\left(\frac{L\left(r,1\right)}{N},\frac{L\left(r,2\right)}{N},\frac{L\left(r,4\right)}{N},\frac{L\left(r,4\right)}{N},\frac{L\left(r,5\right)}{N}\right)\;\;r\in \left\{NE,\;PO,\;HY\right\}$$

where C(r) is the frequency of the r-type amino acids in the sequence, N(r) is the size of the *r^th^* group in an amino acid, *N* is the length of the line, and N(r,s) is the frequency of occurrence of dipeptides from group *rs* to group *sr*; L(r,1), L(r,2), L(r,3), L(r,4) and L(r,5) show information on the location of the *r^th^* group of amino acids in the first 25%, 50%, 75% and 100%. Three classes and seven properties yield 21D (3 × 7) function descriptors in a CTDT or CTDC. The calculations do not account for any gaps.

#### 2.3.3. PAAC and APAAC

The sequence information of AAC and DPC descriptors can be lost, as reported in previous studies [24,25,26]. Chou [25] suggested PAAC and APAAC as solutions to this problem. PAAC takes into account not just the frequency of each amino acid, but also the effect of sequence order on the amino acid sequence [25]. According to Chou, the PAAC is formulated as:

(6) $$\{\begin{array}{c} \theta_{i}=\overset{N-d}{\underset{i=1}{\sum }}\frac{{\left(P_{i}-P_{i+d}\right)}^{2}}{N_{p}}\\ X_{c\left(i\right)}=\frac{N_{i}}{1+\omega \times \sum_{i=1}^{30}\theta_{i}}\\ X_{c_{lambda_{i}}}=\frac{\omega \times \theta_{i}}{1+\omega \times \sum_{i=1}^{30}\theta_{i}} \end{array}$$

where *θ_i_* is the number of factors related to the order of the sequence. *P_i_* is the value of the properties of the *i*-th amino acid. *N_P_* is the number of properties. *N_i_* is the appearance of the *i^th^* amino acid and *ω* is the parameter set to 0.05 here. The APAAC descriptor focuses on the order of amino acids in the sequence [26]. Particularly, APAAC consists of *Pc*(*i*) and *Pcj*(*i*) as defined by Equation (7) where $\tau_{d}$ reflects the sequence-order information. $P_{i}\left(i\right)$ is the value of the *i*-th amino acid for the *j*-th characteristic. The remaining parameters are identical to APAAC. The various trait descriptors represent various aspects of the amino acid sequence’s physicochemical properties.

(7) $$\{\begin{array}{c} \tau_{d}=\frac{\sum_{i=1}^{N-d}P_{i}\left(i\right)\times P_{j}\left(i+d\right)\;}{N-d}\;d=1,2,3,\;\ldots \ldots 30\\ P_{c\left(i\right)}=\frac{N_{i}}{1+\omega \times \sum_{i=1}^{30}\theta_{i}}\\ X_{c_{j}}\left(i\right)=\frac{\omega \times \theta_{i}}{1+\omega \times \sum_{i=1}^{30}\theta_{i}} \end{array}$$

### 2.4. Feature Optimization and Selection

We employed the genetic algorithm based on the self-assessment-report (GA-SAR) algorithm developed by Charoenkwan et al. [27] to select a minimal number of *m* features from a large number of *n* features while simultaneously optimizing the model’s parameters. To date, the GA-SAR has been successfully applied in a number of computational biology studies [27,28,29]. The GA-SAR’s chromosome contains two main genes: (i) binary genes for the feature selection purpose, and (ii) parametric genes for the parameter optimization of SVM classifier. For convenience of discussion, the gene and chromosome will be referred to as GA-gene and GA-chrom, respectively. More details on the GA-SAR algorithm were reported in our previous studies [27,28,29].

### 2.5. Feature Representation Learning Method

Wei et al. [19] were the first to propose the feature representation learning method. Several previous studies [19,28,29,30,31,32,33,34] have found that this method is effective and can improve the model’s discriminative ability. This technique makes a significant contribution in two areas: solving high-dimensional feature space and providing enough information to develop an accurate predictive model. We modified this feature representation learning method by combining multiple ML algorithms in this paper. The procedure of the development of the proposed UMPred-FRL by using the feature representation learning method is described in detail as follows:

*Step 1. Baseline model construction.* We used seven different feature encoding schemes (AAC, APAAC, CTDC, CTDD, CTDT, DPC, and PAAC) derived from three major groups (composition-based features, composition-transition-distribution-based features, and pseudo-amino acid composition-based features). These characteristics were then used to create a set of baseline models using six well-known ML algorithms (ET, KNN, LR, PLS, RF, and SVM). Using the default parameters, 42 baseline models (6 MLs × 7 encodings) were created. All baseline models in this step were created using the Scikit-learn package in Python’s default parameters (version 0.22) [35].

*Step 2. Generation of new feature representations.* All 42 baseline models were trained using a 10-fold cross-validation procedure and then used to generate three types of features containing probabilistic feature (PF), class feature (CF) and the combination of PF and CF (CPF). The PF is based on the predicted probability scores of umami peptides which is in the range of 0–1. In case of the CF, the protein sequence *P* is labeled as 1 (umami peptides) if its predicted probability score is greater than 0.5, otherwise the protein sequence *P* is labeled as 0 (non-umami peptides). As a result, the protein sequence *P* was represented to 42-D, 42-D and 84-D feature vectors for PF, CF, and PCF, respectively. In this study, the PF, CF, and PCF were considered as new feature vectors.

*Step 3. Development of the final meta-predictor.* The final meta-predictor was built individually combining the SVM algorithm (mSVM) with each of the three newly created feature vectors (CF, PF, and CPF). In this process, the GA-SAR algorithm was used to identify informative features of CF, PF, and CPF, followed by simultaneous tuning of the mSVM models’ parameters (C) using a 10-fold cross-validation procedure to improve the discriminative power of the mSVM model (Supplementary Table S1). Herein, the parameter (C ∈ {1, 2, 4, 8, 16, 32}) and *n* features were used as input for optimization via the GA-SAR algorithm. Therefore, the GA-chrom contains *n* binary GA-genes ($f_{i}=1$) for identifying important features and 3-bit GA-genes for determining the *C* parameter. The *i^th^* feature is used for development of the mSVM model where $f_{i}=1;$ otherwise the *i^th^* feature is not used. Finally, the feature set with the highest MCC was chosen as the best and was used to create the final meta-predictor.

### 2.6. Performance Evaluation

We used five commonly used binary classification metrics for performance evaluation: BACC, MCC, Sn, accuracy (ACC), and specificity (Sp) [36]. These metrics are defined as follows:

(8) $$\mathrm{ACC}=\frac{\mathrm{TP}+\mathrm{TN}}{\left(\mathrm{TP}+\mathrm{TN}+\mathrm{FP}+\mathrm{FN}\right)}$$

(9) $$\mathrm{Sn}=\frac{\mathrm{TP}}{\left(\mathrm{TP}+\mathrm{FN}\right)}$$

(10) $$\mathrm{Sp}=\frac{\mathrm{TN}}{\left(\mathrm{TN}+\mathrm{FP}\right)}$$

(11) BACC=(Sn+Sp)×0.5

(12) $$\mathrm{MCC}=\frac{\mathrm{TP}\times \mathrm{TN}-\mathrm{FP}\times \mathrm{FN}}{\sqrt{\left(\mathrm{TP}+\mathrm{FP}\right)\left(\mathrm{TP}+\mathrm{FN}\right)\left(\mathrm{TN}+\mathrm{FP}\right)\left(\mathrm{TN}+\mathrm{FN}\right)}}$$

where TP, TN, FP, and FN represent the number of true positives, true negatives, false positives, and false negatives, respectively. We also plotted receiver operating characteristic (ROC) curves to visualize the overall performance of different models, as well as computing their area under the ROC curve (AUC). The model with the highest AUC was determined to be the best [37,38,39,40,41].

## 3. Results

### 3.1. Performance of Different Baseline Models

**Figure 2:**
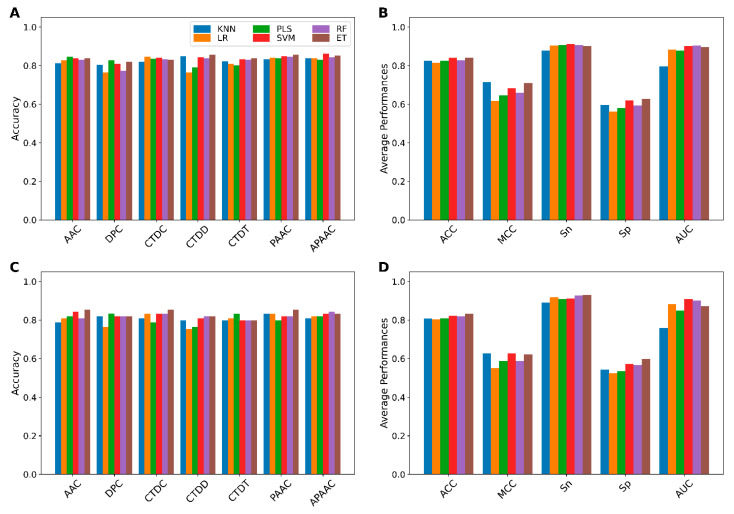
Performance comparison of different baseline models. (**A**,**B**) Cross-validation and (**C**,**D**) independent test results of 42 baseline models. (**A**,**C**) The performance of 42 baseline models in terms of cross-validation and independent test ACC. (**B**,**D**) The average performance of each classifier over seven different feature descriptors on the training and independent test datasets, respectively.

**Figure 3:**
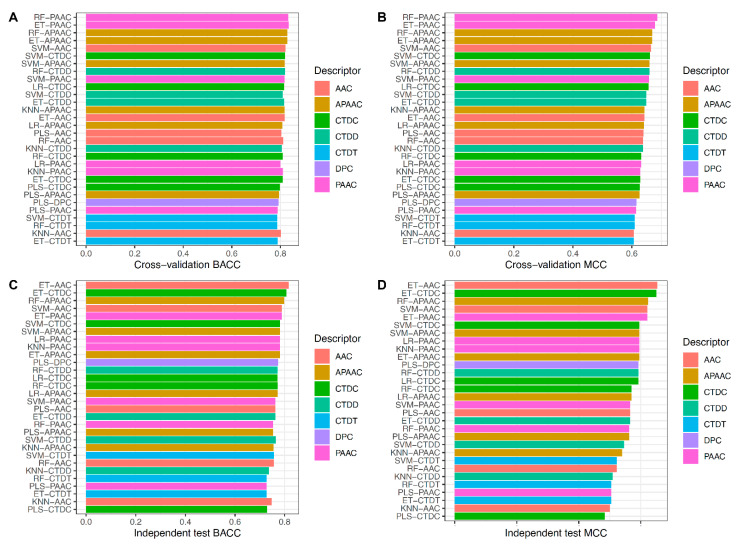
Performance evaluations of top 30 baseline models. (**A**,**B**) Cross-validation BACC and MCC of top 30 baseline models. (**C**,**D**) Independent test BACC and MCC of top 30 baseline models.

We comprehensively compared the performance of 42 baseline models trained using seven different feature-encoding schemes (AAC, APAAC, CTDC, CTDD, CTDT, DPC, and PAAC) with six well-known ML algorithms (ET, KNN, LR, PLS, RF, and SVM) by performing repeated stratified 10-fold cross-validation tests with 10 repetitions. Finally, the average performances obtained from the repeated stratified 10-fold cross-validation scheme were used to determine the best combination of encoding and ML algorithm that were beneficial to umami peptide identification. Results from cross-validation and independent tests are provided in Figure 2 and Figure 3 and Supplementary Tables S2 and S3.

As shown in Figure 2 and Supplementary Table S2, ET, KNN, LR, PLS, RF, and SVM models trained with PAAC, APAAC, CTDC, ACC, PAAC, and AAC descriptors achieved best performances (BACC, MCC) of (0.834, 0.678), (0.818, 0.642), (0.815, 0.657), (0.804, 0.639), (0.832, 0.686), and (0.821, 0.665), respectively. Furthermore, in order to conduct a comparative analysis of the six ML models, the average prediction results of each ML model across the seven feature encodings were calculated and summarized in Figure 2A,C. Particularly, it was found that from amongst the six ML models, ET, RF, and SVM provided the best cross-validation results across all five metrics (i.e., ACC, BACC, Sn, Sp, and MCC). ACC, MCC, and AUC were provided by these three ML models in the ranges of 0.838–0.845, 0.620–0.636 and 0.901–0.911, respectively. Meanwhile, KNN outperformed ET, RF, and SVM with an Sn of 0.715.

In order to select the best baseline model, we examined the prediction results of 42 baseline models using 10-fold cross-validation and independent tests. Figure 3A,B as well as Supplementary Table S3 depict the performance of the 42 baseline models. On the UMP-TR dataset, RF-PAAC and ET-PAAC first-best and second-best baseline models outperformed the other baseline models in four out of six metrics (ACC, BACC, Sn, and MCC). RF-PAAC and ET-PAAC models, in particular, provided maximum ACC, BACC, Sn, and MCC values of 0.864, 0.834, 0.765 and 0.686, respectively. Figure 3C,D as well as Supplementary Table S3 show that RF-PAAC and ET-PAAC models can effectively identify umami peptides with ACC > 0.820, BACC > 0.758, and MCC > 0.563, as evaluated on independent tests. Based on performance comparisons in Figure 2 and Figure 3 as well as Supplementary Tables S2 and S3, the baseline model trained using the RF algorithm and PAAC encoding is considered to be the best baseline model.

### 3.2. Performance of Class, Probabilistic and Fused Information

Instead of making an effort to select the best one from amongst the 42 baseline models, we integrated their individual strengths to develop an ensemble-based model using the meta-predictor approach. Several previous studies have demonstrated that ensemble-based models are able to achieve more accuracy compared with their constituent baseline models [19,28,29,30,32,33,42]. In this study, we employed three different types of new feature representations (CF, PF, and CPF) by training and optimizing three different mSVM models with repeated stratified 10-fold cross-validation tests with 10 repetitions. Table 1 and Table 2 show the results of their cross-validation and independent tests. As shown in Table 1, PF outperforms CF and CPF in four out of six metrics (ACC, BACC, Sp, and MCC). Particularly, the ACC, BACC, Sp, and MCC of PF were 0.860, 0.830, 0.914, and 0.677, respectively. To improve the predictive ability of our feature representation, the GA-SAR algorithm was used to individually determine the optimal features on each of the three feature vectors. Finally, the GA-SAR algorithm identified 10, 7, and 8 informative features for CF, PF and CPF, respectively.

We observed significant improvements in the optimal features of PF by comparing the predictive performance of the original (42D) and newly informative (7D) features, achieving 3.7%, 4.0%, 4.9%, 3.2%, 8.8%, and 3.0% improvements in terms of ACC, BACC, Sn, SP, MCC, and AUC (Table 1). Surprisingly, the 7 informative features of PF also had the best predictive performance when compared to the best features of CF and CPF. In this paper, the 7 baseline models of SVM-AAC, PLS-AAC, SVM-CTDC, RF-DPC, RF-CTDC, PLS-APAAC and LR-DPC were used to generate the 7 informative features of PF. In the case of independent test results, we discovered that the optimal PF features performed slightly better than the optimal CF and CPF features (Table 2). Taking both cross-validation and independent test results into account, our new feature representations (the 7 informative features of PF) demonstrated stable performance on both training and independent datasets and were deemed the best feature set to develop the final meta-predictor (termed UMPred-FRL).

### 3.3. New Feature Representations Improve the Prediction Performance

We examined the efficacy of our new feature representations by comparing their performance to that of seven different feature descriptors (AAC, APAAC, CTDC, CTDD, CTDT, DPC, and PAAC). In particular, we used an SVM classifier to train and evaluate the seven feature descriptors in order to compare them fairly with our new feature representations. Table 3 and Table 4 show the results of their cross-validation and independent tests, respectively.

**Figure 4:**
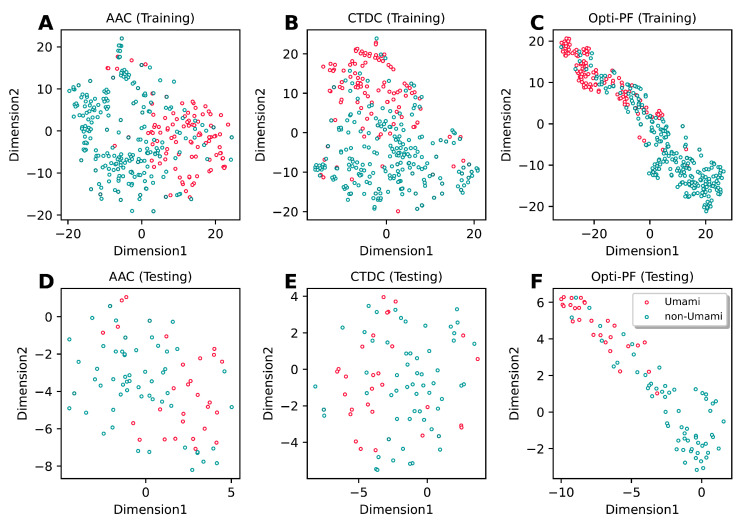
t-distributed stochastic neighbor embedding (t-SNE) distribution of the positive and negative samples on the training (**A**–**C**) and independent test (**D**–**F**) datasets, respectively. (**A**,**D**) AAC, (**B**,**E**) CTDC and (**C**,**F**) optimal PF.

From Table 3, it can be clearly seen that our new feature representations exhibited the best overall cross-validation performance in terms of five out of six metrics (ACC, BACC, Sn, MCC, and AUC). In particular, the ACC, BACC, Sn, and MCC of our feature representations were 4.2–8.6%, 5.0–13.6%, 6.8–27.5% and 10.0–21.6%, respectively, higher than those of other descriptors. Furthermore, our feature representations’ independent test results exhibited a similar tendency to the cross-validation results (Table 4). To confirm the discriminative power of our feature representations, the feature space distribution was compared with the top two feature descriptors (AAC and CTDC) using the t-distributed stochastic neighbor embedding (t-SNE). Therefore, t-SNE plots were created for both the training and independent test datasets in this study. Figure 4 depicts the distribution of the feature space in a 2D feature space between umami (red spots) and non-umami (green spots) peptides. As shown in Figure 4C,F, the feature space of our feature representations showed a more distinct separation of the margins between two clusters than AAC (Figure 4A,D) and CTDC (Figure 4B,E) descriptors. This demonstrated that our feature representation learnings outperformed conventional feature descriptors in terms of discriminative power.

### 3.4. Mechanistic Interpretation of UMPred-FRL

**Figure 5:**
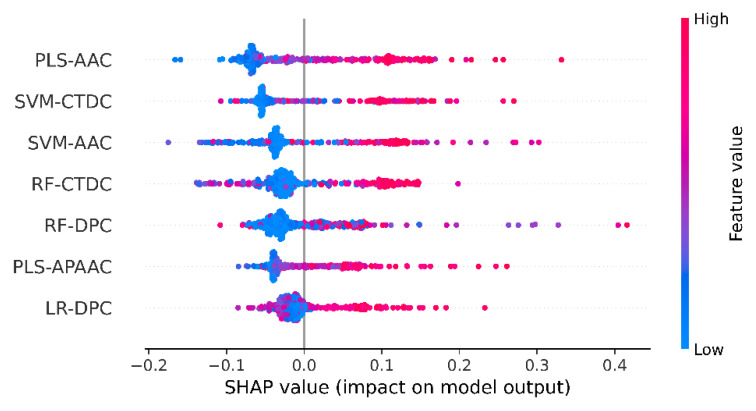
SHAP values of informative 7 probabilistic features used for UMPred-FRL. SHAP values represent the directionality of the informative features, where positive and negative SHAP values represent positive (umami peptide) and negative (non-umami peptide) predictions.

To determine which features were the most important for the proposed UMPred-FRL and its constituent baseline models, we applied the SHapley Additive exPlanation (SHAP) approach. The SHAP method has been widely utilized to improve interpretable predictions and measure the features’ value for the predictions of complex ML models, such as ensemble or deep learning models [43,44]. It should be noted that positive and negative SHAP values drive the predictions towards umami and non-umami peptides, respectively. As can be seen from Figure 5, the top three PFs consist of three baseline models of PLS-AAC, SVM-AAC, and SVM-CTDC. It became apparent that when the top three PFs had high values, their corresponding SHAP values would also positively influence the model’s prediction of umami peptides. Similarly, by taking into consideration the feature importance from PLS-AAC and SVM-AAC, it was found that Glu and Asp were the top two informative features that exhibited positive SHAP values (Supplementary Figure S1), thereby indicating that Glu and Asp might be crucial factors responsible for umami tastes.

### 3.5. Comparison of UMPred-FRL with Its Constituent Baseline Models and the Existing Method

**Figure 6:**
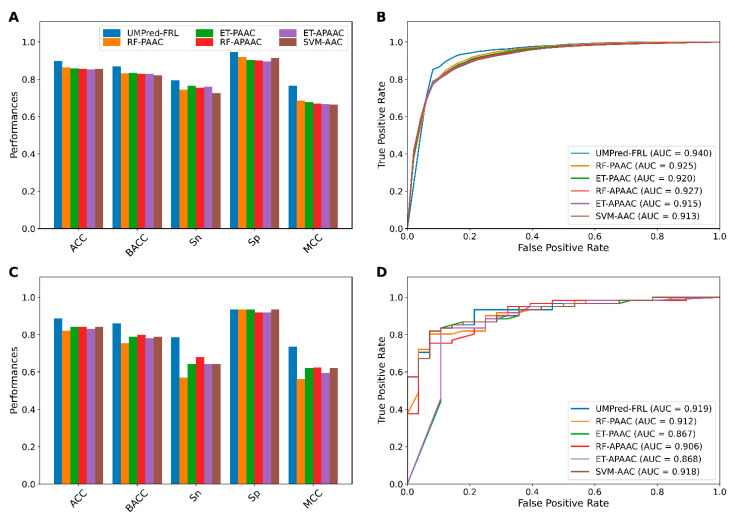
Performance comparison of UMPred-FRL with the top five baseline models on the training (**A**,**B**) and independent test (**C**,**D**) datasets. Prediction results of UMPred-FRL and the top five baseline models in terms of ACC, BACC, Sn, Sp, and MCC. (**C**,**D**) ROC curves and AUC values of the top five baseline models.

To assess the efficacy and robustness of the proposed UMPred-FRL, we first compared it to the top five baseline models with the highest cross-validation MCC (RF-PAAC, ET-PAAC, RF-APAAC, ET-APAAC, and SVM-AAC). Cross-validation results (Figure 6A,B) show that UMPred-FRL clearly outperforms the top five baseline models in terms of all six metrics, achieving 3.4–4.4%, 3.6–4.9%, 3.0–6.8%, 2.5–4.9%, 7.9–10.0%, and 1.5–2.7% improvements in ACC, BACC, Sn, Sp, MCC, and AUC, respectively. UM-Pred-FRL also performed admirably in the independent test when compared to the top five baseline models. In particular, ACC, BACC, Sn, and MCC of UMPred-FRL were 4.5–6.8%, 6.2–10.7%, 10.7–21.5%, and 11.1–17.2% higher than those of other baseline models, respectively (Figure 6C,D).

**Figure 7:**
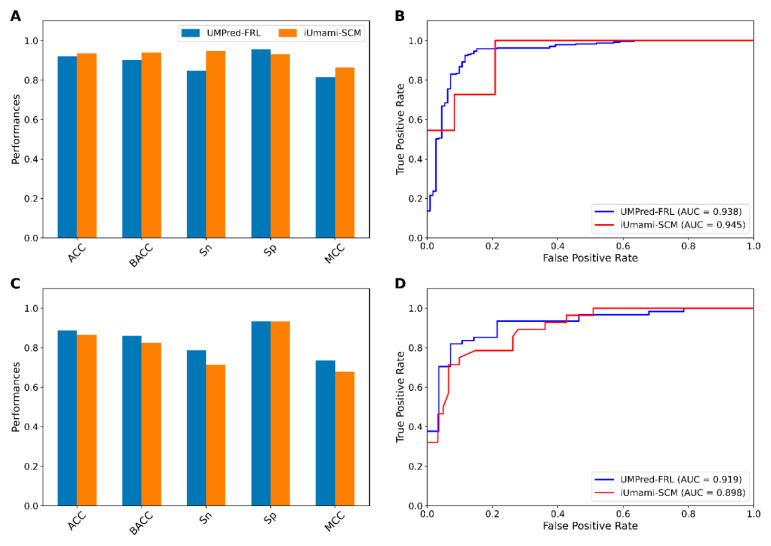
Performance of the proposed UMPred-FRL and the existing method (iUmami-SCM) on training (**A**,**B**) and independent test (**C**,**D**) datasets. (**A**,**B**) Prediction results of UMPred-FRL and iUmami-SCM in terms of ACC, BACC, Sn, Sp and MCC. (**C**,**D**) ROC curves and AUC values of UMPred-FRL and iUmami-SCM.

To demonstrate the robustness of UMPred-FRL, its performance was compared to that of the existing method (iUmami-SCM). As such, the 10-fold cross-validation and independent test results are shown in Figure 7 and Table 5. On the UMP-TR dataset, as shown in Figure 7A,B, UMPred-FRL achieves very comparable performance (ACC, BACC, and AUC) to iUmami-SCM (0.921 vs. 0.935, 0.901 vs. 0.939, and 0.938 vs. 0.945, respectively). On the other hand, it was clear that UMPred-FRL could outperform iUmami-SCM in five out of six metrics on the independent test dataset (Figure 7C,D). In particular, BACC, Sn, and MCC of UMPred-FRL were 3.6%, 7.2%, and 5.6%, respectively, higher than the corresponding values afforded by iUmami-SCM. Remarkably, the outstanding Sn and MCC indicated that the proposed UMPred-FRL is capable of eliminating the number of false negatives and false positives on unknown samples (Table 5). Taken together, comparative results indicated that UMPred-FRL is more effective than, and could outperform, the existing method as well as its constituent baseline models for the identification of umami peptides.

## 4. Conclusions

In this study, we developed UMPred-FRL, a novel machine-learning meta-predictor for the accurate identification of umami peptides based on sequence information and without knowledge of the protein’s 3D structure. UMPred-FRL built 42 baseline models by exploring six different ML classifiers with seven different feature encodings using the feature representation learning method. These baseline models were then used to generate predicted probabilistic scores of umami peptides, which were considered as new feature representations. Finally, the resulting features were combined and chosen in order to create a more stable meta-predictor based on the SVM algorithm. Our cross-validation and independent test results demonstrated the efficacy and robustness of UMPred-FRL by outperforming its constituent baseline models. Furthermore, on the independent test dataset, UMPred-FRL consistently outperformed the existing method (iUmami-SCM) in terms of BACC (0.860 vs. 0.824), Sn (0.786 vs. 0.714), and MCC (0.735 vs. 0.679), highlighting its effectiveness and generalizability. We discovered that our new feature representations were more discriminative in capturing the key information of umami peptides when compared to seven well-known feature encodings. Finally, in order to maximize the utility of our proposed predictor, we set up a publicly accessible web server at http://pmlabstack.pythonanywhere.com/UMPred-FRL (accessed on 1 December 2021). It is anticipated that UMPred-FRL will be a powerful tool for the discovery of candidate peptides with potential umami sensory properties as well as the characterization of umami peptide mechanisms.

## Figures and Tables

**Table 1:** Cross-validation results for CF, PF, CFP and their optimal sets.

Feature Set	Dimension	ACC	BACC	Sn	Sp	MCC	AUC
CF	42	0.854	0.823	0.741	0.906	0.662	0.903
PF	42	0.860	0.830	0.746	0.914	0.677	0.909
CPF	84	0.855	0.827	0.749	0.905	0.667	0.915
Optimal CF	10	0.875	0.870	0.857	0.884	0.729	0.887
Optimal PF	7	0.898	0.870	0.795	0.945	0.765	0.940
Optimal CPF	8	0.893	0.868	0.797	0.938	0.755	0.895

**Table 2:** Independent test results for CF, PF, CFP and their optimal sets.

Feature Set	Dimension	ACC	BACC	Sn	Sp	MCC	AUC
CF	42	0.876	0.842	0.750	0.934	0.707	0.934
PF	42	0.820	0.763	0.607	0.918	0.565	0.930
CPF	84	0.798	0.698	0.429	0.967	0.505	0.934
Optimal CF	10	0.876	0.881	0.893	0.869	0.732	0.904
Optimal PF	7	0.888	0.860	0.786	0.934	0.735	0.919
Optimal CPF	8	0.888	0.870	0.821	0.918	0.739	0.898

**Table 3:** Cross-validation results of new feature representations and conventional feature descriptors.

Feature	Dimension	ACC	BACC	Sn	Sp	MCC	AUC
AAC	20	0.856	0.821	0.727	0.915	0.665	0.913
APAAC	22	0.854	0.818	0.719	0.917	0.660	0.917
CTDC	39	0.854	0.820	0.727	0.912	0.661	0.911
CTDD	195	0.850	0.810	0.700	0.920	0.649	0.914
CTDT	39	0.834	0.786	0.655	0.917	0.609	0.875
DPC	400	0.812	0.734	0.520	0.947	0.549	0.892
PAAC	21	0.854	0.818	0.719	0.916	0.658	0.919
Optimal PF	7	0.898	0.870	0.795	0.945	0.765	0.940

**Table 4:** Independent test results of new feature representations and conventional feature descriptors.

Feature	Dimension	ACC	BACC	Sn	Sp	MCC	AUC
AAC	20	0.843	0.789	0.643	0.934	0.621	0.918
APAAC	22	0.831	0.780	0.643	0.918	0.595	0.923
CTDC	39	0.831	0.780	0.643	0.918	0.595	0.923
CTDD	195	0.809	0.764	0.643	0.885	0.546	0.894
CTDT	39	0.798	0.756	0.643	0.869	0.523	0.872
DPC	400	0.798	0.708	0.464	0.951	0.502	0.908
PAAC	21	0.820	0.763	0.607	0.918	0.565	0.924
Optimal PF	7	0.888	0.860	0.786	0.934	0.735	0.919

**Table 5:** Cross-validation and independent test results of UMPred-FRL and the existing method.

Cross-Validation	Method	ACC	BACC	Sn	Sp	MCC	AUC
10-fold CV	iUmami-SCM	0.935	0.939	0.947	0.930	0.864	0.945
	UMPred-FRL	0.921	0.901	0.847	0.955	0.814	0.938
Independent test	iUmami-SCM	0.865	0.824	0.714	0.934	0.679	0.898
	UMPred-FRL	0.888	0.860	0.786	0.934	0.735	0.919

## Data Availability

All the data are available at http://pmlabstack.pythonanywhere.com/UMPred-FRL (accessed on 1 December 2021).

## Supplementary Materials

The following are available online at https://www.mdpi.com/article/10.3390/ijms222313124/s1.
