# Supplementary material for: UMPred-FRL: A New Approach for Accurate Prediction of Umami Peptides Using Feature Representation Learning
Source: Int J Mol Sci. 2021 Dec 4;22(23):13124. doi: 10.3390/ijms222313124 (PMC8658322; doi:10.3390/ijms222313124)
Supplement: Supplementary file 1 [file ijms-22-13124-s001.zip › ijms-1457796-supplementary.pdf]

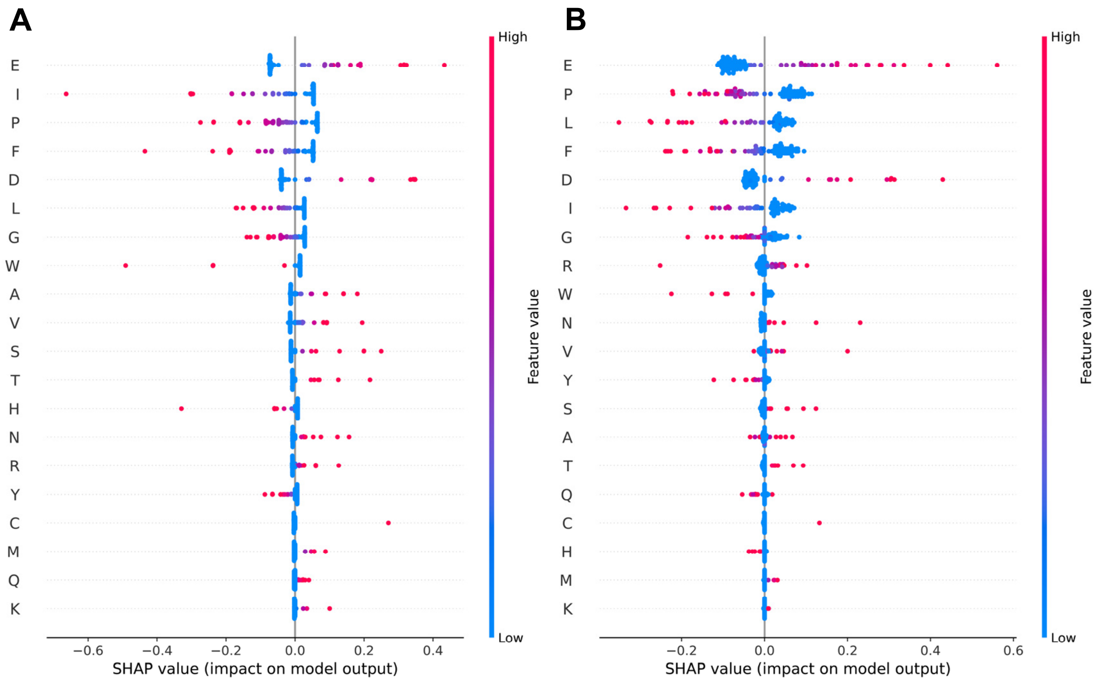

Figure S1. SHAP values of PLS-AAC **(A)** and SVM-AAC **(B)**. SHAP values represent the directionality of the informative features, where positive and negative SHAP values represent positive (umami peptides) and negative (non-umami peptides) predictions

Table S1. Hyperparameter search details for five different ML classifiers.

Method	Parameters	Range of parameters
KNN	number of neighbours	Default
MLP	hidden_layer_sizes	[50, 100, 300, 500]
ET	n_estimators	[20, 50, 100, 200, 500]
RF	n_estimators	[20, 50, 100, 200, 500]
	max_features	sqrt(n_features)
SVM	penalty parameter (C[a])	[1, 2, 4, 8, 16, 32]
	kernel coefficient (γ[a])	Default

Columns 2 and 3 represents the parameter name used in the Scikit-learn library and the range of parameter used to develop the model, respectively.

Table S2 Cross-validation results of diffident baseline models developed using six different ML algorithms and seven feature descriptors.

Feature	Model	ACC	BACC	Sn	Sp	MCC	AUC
AAC	ET	0.842 (0.038)	0.818 (0.039)	0.751 (0.045)	0.885 (0.033)	0.642 (0.050)	0.904 (0.031)
	KNN	0.827 (0.039)	0.802 (0.041)	0.732 (0.046)	0.871 (0.035)	0.607 (0.051)	0.802 (0.042)
	LR	0.834 (0.039)	0.778 (0.038)	0.623 (0.051)	0.933 (0.026)	0.605 (0.051)	0.924 (0.028)
	PLS	0.846 (0.038)	0.804 (0.038)	0.687 (0.048)	0.920 (0.028)	0.639 (0.050)	0.919 (0.029)
	RF	0.843 (0.038)	0.812 (0.039)	0.726 (0.047)	0.898 (0.032)	0.638 (0.050)	0.919 (0.029)
	SVM	0.856 (0.037)	0.821 (0.038)	0.727 (0.046)	0.915 (0.029)	0.665 (0.049)	0.913 (0.029)
APAAC	ET	0.854 (0.037)	0.829 (0.038)	0.762 (0.044)	0.896 (0.032)	0.668 (0.049)	0.915 (0.029)
	KNN	0.844 (0.038)	0.818 (0.039)	0.746 (0.045)	0.889 (0.033)	0.642 (0.050)	0.818 (0.040)
	LR	0.845 (0.038)	0.809 (0.039)	0.708 (0.047)	0.909 (0.030)	0.641 (0.050)	0.914 (0.029)
	PLS	0.841 (0.038)	0.794 (0.039)	0.666 (0.049)	0.922 (0.028)	0.626 (0.050)	0.915 (0.029)
	RF	0.855 (0.037)	0.829 (0.038)	0.755 (0.045)	0.902 (0.031)	0.669 (0.049)	0.927 (0.027)
	SVM	0.854 (0.037)	0.818 (0.038)	0.719 (0.047)	0.917 (0.029)	0.660 (0.049)	0.917 (0.029)
CTDC	ET	0.836 (0.039)	0.810 (0.040)	0.739 (0.046)	0.881 (0.034)	0.628 (0.050)	0.894 (0.032)
	KNN	0.824 (0.040)	0.800 (0.041)	0.733 (0.046)	0.866 (0.036)	0.602 (0.051)	0.800 (0.042)
	LR	0.852 (0.037)	0.815 (0.038)	0.715 (0.047)	0.915 (0.029)	0.657 (0.050)	0.924 (0.028)
	PLS	0.841 (0.038)	0.797 (0.039)	0.676 (0.049)	0.918 (0.029)	0.627 (0.050)	0.909 (0.030)
	RF	0.839 (0.038)	0.809 (0.039)	0.727 (0.046)	0.891 (0.032)	0.631 (0.050)	0.912 (0.030)
	SVM	0.854 (0.037)	0.820 (0.038)	0.727 (0.046)	0.912 (0.030)	0.661 (0.049)	0.911 (0.030)
CTDD	ET	0.847 (0.038)	0.815 (0.039)	0.727 (0.046)	0.903 (0.031)	0.648 (0.050)	0.918 (0.029)
	KNN	0.845 (0.038)	0.806 (0.039)	0.698 (0.048)	0.913 (0.029)	0.638 (0.050)	0.806 (0.041)
	LR	0.775 (0.044)	0.749 (0.044)	0.677 (0.049)	0.821 (0.040)	0.499 (0.052)	0.803 (0.042)

	PLS	0.792 (0.042)	0.732 (0.042)	0.567 (0.052)	0.897 (0.032)	0.502 (0.052)	0.838 (0.038)
	RF	0.853 (0.037)	0.820 (0.038)	0.729 (0.046)	0.910 (0.030)	0.659 (0.049)	0.915 (0.029)
	SVM	0.850 (0.037)	0.810 (0.038)	0.700 (0.048)	0.920 (0.028)	0.649 (0.050)	0.914 (0.029)
CTDT	ET	0.833 (0.039)	0.789 (0.040)	0.668 (0.049)	0.909 (0.030)	0.607 (0.051)	0.877 (0.034)
	KNN	0.816 (0.040)	0.789 (0.041)	0.713 (0.047)	0.864 (0.036)	0.579 (0.052)	0.789 (0.043)
	LR	0.815 (0.041)	0.764 (0.041)	0.625 (0.050)	0.902 (0.031)	0.561 (0.052)	0.855 (0.037)
	PLS	0.799 (0.042)	0.740 (0.041)	0.576 (0.052)	0.903 (0.031)	0.521 (0.052)	0.860 (0.036)
	RF	0.834 (0.039)	0.787 (0.039)	0.657 (0.050)	0.916 (0.029)	0.609 (0.051)	0.893 (0.032)
	SVM	0.834 (0.039)	0.786 (0.039)	0.655 (0.050)	0.917 (0.029)	0.609 (0.051)	0.875 (0.035)
DPC	ET	0.812 (0.041)	0.743 (0.039)	0.554 (0.052)	0.931 (0.026)	0.548 (0.052)	0.876 (0.034)
	KNN	0.813 (0.041)	0.768 (0.041)	0.645 (0.050)	0.891 (0.032)	0.560 (0.052)	0.768 (0.044)
	LR	0.756 (0.045)	0.619 (0.026)	0.242 (0.045)	0.995 (0.008)	0.392 (0.051)	0.895 (0.032)
	PLS	0.836 (0.039)	0.793 (0.039)	0.673 (0.049)	0.912 (0.030)	0.615 (0.051)	0.809 (0.041)
	RF	0.776 (0.043)	0.668 (0.035)	0.371 (0.050)	0.965 (0.019)	0.448 (0.052)	0.887 (0.033)
	SVM	0.812 (0.041)	0.734 (0.038)	0.520 (0.052)	0.947 (0.023)	0.549 (0.052)	0.892 (0.032)
PAAC	ET	0.859 (0.036)	0.834 (0.038)	0.765 (0.044)	0.903 (0.031)	0.678 (0.049)	0.920 (0.028)
	KNN	0.837 (0.038)	0.811 (0.040)	0.738 (0.046)	0.883 (0.033)	0.629 (0.050)	0.811 (0.041)
	LR	0.842 (0.038)	0.800 (0.039)	0.685 (0.048)	0.914 (0.029)	0.631 (0.050)	0.913 (0.029)
	PLS	0.836 (0.039)	0.788 (0.039)	0.658 (0.049)	0.918 (0.029)	0.614 (0.051)	0.912 (0.030)
	RF	0.864 (0.036)	0.832 (0.037)	0.744 (0.046)	0.920 (0.028)	0.686 (0.048)	0.925 (0.027)
	SVM	0.854 (0.037)	0.818 (0.038)	0.719 (0.047)	0.916 (0.029)	0.658 (0.049)	0.919 (0.029)

Table S3 Independent test results of diffident baseline models developed using six different ML algorithms and seven feature descriptors.

Feature	Model	ACC	BACC	Sn	Sp	MCC	AUC
AAC	ET	0.854 (0.073)	0.816 (0.075)	0.714 (0.094)	0.918 (0.057)	0.653 (0.099)	0.864 (0.071)
	KNN	0.787 (0.085)	0.748 (0.087)	0.643 (0.100)	0.852 (0.074)	0.500 (0.104)	0.748 (0.090)
	LR	0.809 (0.082)	0.735 (0.078)	0.536 (0.104)	0.934 (0.051)	0.533 (0.104)	0.912 (0.059)
	PLS	0.820 (0.080)	0.763 (0.079)	0.607 (0.101)	0.918 (0.057)	0.565 (0.103)	0.913 (0.059)
	RF	0.798 (0.083)	0.756 (0.085)	0.643 (0.100)	0.869 (0.070)	0.523 (0.104)	0.910 (0.060)
	SVM	0.843 (0.076)	0.789 (0.075)	0.643 (0.100)	0.934 (0.051)	0.621 (0.101)	0.918 (0.057)
APAAC	ET	0.831 (0.078)	0.780 (0.078)	0.643 (0.100)	0.918 (0.057)	0.595 (0.102)	0.868 (0.070)
	KNN	0.809 (0.082)	0.754 (0.082)	0.607 (0.101)	0.902 (0.062)	0.540 (0.104)	0.754 (0.089)
	LR	0.820 (0.080)	0.772 (0.081)	0.643 (0.100)	0.902 (0.062)	0.570 (0.103)	0.907 (0.060)
	PLS	0.820 (0.080)	0.753 (0.077)	0.571 (0.103)	0.934 (0.051)	0.563 (0.103)	0.901 (0.062)
	RF	0.843 (0.076)	0.798 (0.077)	0.679 (0.097)	0.918 (0.057)	0.624 (0.101)	0.906 (0.061)
	SVM	0.831 (0.078)	0.780 (0.078)	0.643 (0.100)	0.918 (0.057)	0.595 (0.102)	0.923 (0.055)
CTDC	ET	0.854 (0.073)	0.806 (0.074)	0.679 (0.097)	0.934 (0.051)	0.650 (0.099)	0.864 (0.071)
	KNN	0.809 (0.082)	0.754 (0.082)	0.607 (0.101)	0.902 (0.062)	0.540 (0.104)	0.754 (0.089)
	LR	0.831 (0.078)	0.771 (0.076)	0.607 (0.101)	0.934 (0.051)	0.592 (0.102)	0.907 (0.060)
	PLS	0.787 (0.085)	0.728 (0.085)	0.571 (0.103)	0.885 (0.066)	0.484 (0.104)	0.857 (0.073)
	RF	0.820 (0.080)	0.772 (0.081)	0.643 (0.100)	0.902 (0.062)	0.570 (0.103)	0.907 (0.060)
	SVM	0.831 (0.078)	0.780 (0.078)	0.643 (0.100)	0.918 (0.057)	0.595 (0.102)	0.923 (0.055)
CTDD	ET	0.820 (0.080)	0.763 (0.079)	0.607 (0.101)	0.918 (0.057)	0.565 (0.103)	0.898 (0.063)
	KNN	0.798 (0.083)	0.737 (0.082)	0.571 (0.103)	0.902 (0.062)	0.509 (0.104)	0.737 (0.092)
	LR	0.753 (0.090)	0.694 (0.089)	0.536 (0.104)	0.852 (0.074)	0.406 (0.102)	0.862 (0.072)

	PLS	0.764 (0.088)	0.712 (0.088)	0.571 (0.103)	0.852 (0.074)	0.438 (0.103)	0.757 (0.089)
	RF	0.831 (0.078)	0.771 (0.076)	0.607 (0.101)	0.934 (0.051)	0.592 (0.102)	0.909 (0.060)
	SVM	0.809 (0.082)	0.764 (0.083)	0.643 (0.100)	0.885 (0.066)	0.546 (0.103)	0.894 (0.064)
CTDT	ET	0.798 (0.083)	0.727 (0.080)	0.536 (0.104)	0.918 (0.057)	0.505 (0.104)	0.847 (0.075)
	KNN	0.798 (0.083)	0.775 (0.085)	0.714 (0.094)	0.836 (0.077)	0.541 (0.104)	0.775 (0.087)
	LR	0.809 (0.082)	0.764 (0.083)	0.643 (0.100)	0.885 (0.066)	0.546 (0.103)	0.806 (0.082)
	PLS	0.831 (0.078)	0.780 (0.078)	0.643 (0.100)	0.918 (0.057)	0.595 (0.102)	0.811 (0.081)
	RF	0.798 (0.083)	0.727 (0.080)	0.536 (0.104)	0.918 (0.057)	0.505 (0.104)	0.863 (0.072)
	SVM	0.798 (0.083)	0.756 (0.085)	0.643 (0.100)	0.869 (0.070)	0.523 (0.104)	0.872 (0.069)
DPC	ET	0.843 (0.076)	0.789 (0.075)	0.643 (0.100)	0.934 (0.051)	0.621 (0.101)	0.897 (0.063)
	KNN	0.820 (0.080)	0.763 (0.079)	0.607 (0.101)	0.918 (0.057)	0.565 (0.103)	0.763 (0.088)
	LR	0.764 (0.088)	0.625 (0.045)	0.250 (0.090)	1.000 (0.000)	0.431 (0.103)	0.885 (0.066)
	PLS	0.833 (0.077)	0.773 (0.077)	0.615 (0.101)	0.931 (0.053)	0.593 (0.102)	0.808 (0.082)
	RF	0.820 (0.080)	0.724 (0.065)	0.464 (0.104)	0.984 (0.026)	0.571 (0.103)	0.899 (0.063)
	SVM	0.798 (0.083)	0.708 (0.074)	0.464 (0.104)	0.951 (0.045)	0.502 (0.104)	0.908 (0.060)
PAAC	ET	0.843 (0.076)	0.789 (0.075)	0.643 (0.100)	0.934 (0.051)	0.621 (0.101)	0.867 (0.070)
	KNN	0.831 (0.078)	0.780 (0.078)	0.643 (0.100)	0.918 (0.057)	0.595 (0.102)	0.780 (0.086)
	LR	0.831 (0.078)	0.780 (0.078)	0.643 (0.100)	0.918 (0.057)	0.595 (0.102)	0.904 (0.061)
	PLS	0.798 (0.083)	0.727 (0.080)	0.536 (0.104)	0.918 (0.057)	0.505 (0.104)	0.899 (0.063)
	RF	0.820 (0.080)	0.753 (0.077)	0.571 (0.103)	0.934 (0.051)	0.563 (0.103)	0.912 (0.059)
	SVM	0.820 (0.080)	0.763 (0.079)	0.607 (0.101)	0.918 (0.057)	0.565 (0.103)	0.924 (0.055)
